# EMDomics: a robust and powerful method for the identification of genes differentially expressed between heterogeneous classes

**DOI:** 10.1093/bioinformatics/btv634

**Published:** 2015-10-29

**Authors:** Sheida Nabavi, Daniel Schmolze, Mayinuer Maitituoheti, Sadhika Malladi, Andrew H. Beck

**Affiliations:** ^1^Center for Biomedical Informatics, Harvard Medical School, Boston, MA, USA,; ^2^Department of Pathology and Cancer Research Institute, Beth Israel Deaconess Medical Center and Harvard Medical School, Boston, MA, USA,; ^3^Department of Medicine, Massachusetts General Hospital and Harvard Medical School, Boston, MA, USA and; ^4^The Harker School, San Jose, CA, USA

## Abstract

**Motivation:** A major goal of biomedical research is to identify molecular features associated with a biological or clinical class of interest. Differential expression analysis has long been used for this purpose; however, conventional methods perform poorly when applied to data with high within class heterogeneity.

**Results:** To address this challenge, we developed EMDomics, a new method that uses the Earth mover’s distance to measure the overall difference between the distributions of a gene’s expression in two classes of samples and uses permutations to obtain *q-*values for each gene. We applied EMDomics to the challenging problem of identifying genes associated with drug resistance in ovarian cancer. We also used simulated data to evaluate the performance of EMDomics, in terms of sensitivity and specificity for identifying differentially expressed gene in classes with high within class heterogeneity. In both the simulated and real biological data, EMDomics outperformed competing approaches for the identification of differentially expressed genes, and EMDomics was significantly more powerful than conventional methods for the identification of drug resistance-associated gene sets. EMDomics represents a new approach for the identification of genes differentially expressed between heterogeneous classes and has utility in a wide range of complex biomedical conditions in which sample classes show within class heterogeneity.

**Availability and implementation:** The R package is available at http://www.bioconductor.org/packages/release/bioc/html/EMDomics.html

**Contact:** abeck2@bidmc.harvard.edu

**Supplementary information:** supplementary data are available at *Bioinformatics* online.

## 1 Introduction

Genomic methods enable the measurement of tens-of-thousands to millions of molecular analytes in parallel from a single sample. A major goal of biomedical research is to use these technologies to identify molecular features (e.g. gene-expression patterns) associated with a biological or clinical class of interest, leading to improved understanding of disease pathogenesis and the development of improved diagnostics and therapeutics. Statistical methods have been developed for supervised analyses of gene expression profiling data [e.g. significance analysis of microarrays (SAM) (Tusher *et al.*, 2001), Limma (Smyth, 2004), cuffdiff (Trapnell *et al.*, 2012), DESeq (Anders and Huber, 2010) and edgeR (Robinson *et al.*, 2010)], and these methods (and variants of them) are widely used in the genomics community for the identification of genes differentially expressed between classes.

In general, these methods use a variant of the *t*-statistic (e.g. SAM, Limma, cuffdiff) or approaches designed specifically for sequence read count data (such as that obtained by RNA-Seq) based on an assumption of distribution of aligned short reads (such as negative binomial distribution) to generate a test statistic to summarize the gene’s differential expression. These approaches then use a statistical test (e.g. Fisher exact test for edgeR and DESeq) or permutation tests (e.g. SAM) to determine statistical significance of the differential expression. For *t*-test-based methods, a gene’s test score and significance will tend to be high when the difference in the mean of the gene’s expression levels between the classes is large and the gene’s variance is low. Thus, these methods perform well for the identification of genes that show a high degree of between classes (inter-class) heterogeneity in expression and a low degree of within class (intra-class) heterogeneity. For the Fisher exact test based methods, the null hypothesis is that there is no association between a gene’s read counts and sample class labels. Similar to the *t*-test-based methods, these methods do not effectively capture intra-class heterogeneity since all expression values are summed within a group to calculate the test statistic. In addition, because these methods are based on parametric models, they do not perform well when the data do not fit the method’s model, limiting their generalizability.

Although the term *differentially expressed* is now widely used in the genomics community to refer to a gene that shows a significant difference in mean expression between two classes, difference in mean expression is not the only way a gene may be expressed differentially between two classes, and conventional approaches fail to capture alternative types of differential expression (e.g. two classes with identical means and variances, but samples in one class show a bimodal pattern of expression versus the other class which shows a unimodal pattern of expression across samples). We expect this limitation of conventional approaches to be especially important for the identification of genes associated with phenotypic classes showing significant intraclass heterogeneity. For example, a major goal of genomics research in cancer is to identify genes associated with drug resistance. Given the significant inter-patient molecular heterogeneity in cancer (Burrell *et al.*, 2013) (in which subtypes of tumors are driven by distinct sets of molecular alterations), it is likely that among both sensitive and resistant tumors, there is heterogeneity in the molecular factors driving the sensitive or resistant phenotype in each class. In most cases, the structure of this intraclass heterogeneity will not be known ahead of time; thus, this unknown subtype structure cannot be incorporated into standard supervised statistical methods, which will limit the power of conventional methods for identification of the relevant genes.

In a broader sense, the problems that sample heterogeneity impose on analyzing gene expression data have been previously recognized and several methods to address these problems have been proposed. For example, the cMonkey algorithm (Reiss *et al.*, 2006) has been proposed to detect putative coregulated gene groupings by clustering on both genes and conditions and integrating functional associations and detection of sequence motifs to overcome heterogeneity across samples and conditions.

In particular, several prior methods have been proposed as alternatives to conventional differential expression analysis for prioritizing genes, which may show heterogeneity both within and across groups of samples. Lyons-Weiler *et al.* proposed a heuristic method based on counting the number of samples in both groups that are found beyond the *n*^th^ percentile of the samples in the opposite group to identify genes that have different pattern of expression in the two groups, beside difference in means. This method is called permutation percentile separability test (PPST) (Lyons-Weiler *et al.*, 2004). MacDonald and Ghosh proposed a method, Cancer Outlier Profile Analysis (COPA), based on robust centering and scaling of the data to identify pairs of samples with mutually exclusive outliers, and they used this approach to identify genes involved in recurrent translocations (MacDonald and Ghosh, 2006). Both of these methods may improve on conventional differential expression analyses for special cases of intra-group heterogeneity [e.g. identification of genes that show differential patterns of high- and low-expression across groups according to a pre-specified percentile threshold (Lyons-Weiler *et al.*, 2004) and prioritization of pairs of outlier genes (MacDonald and Ghosh, 2006)].

The primary goal of our study is to develop a powerful, robust, and general approach for the identification of genes differentially expressed between classes that have significant intraclass heterogeneity. To achieve this goal, we compare distribution functions of expression values between classes instead of considering just the first few moments of the distributions (such as mean and variance) or using parametric models. To do this, we use the Earth mover’s distance (EMD) (Rubner *et al.*, 1998, 2000), which is an approach commonly used in image processing to compute distances between color histograms of two digital images. There are several other methods for distribution comparisons. In this work, we chose EMD because it is a nonparametric method, is not sensitive to histogram binning, gives a measure of ground distance, allows for partial matches, and can be computed efficiently (Rubner *et al.*, 2000). To our knowledge, EMD has not previously been developed or tested for differential gene expression analysis. In the setting of two-class problems, we use EMD to measure the overall difference between the distributions of a gene’s expression in two classes of samples. After computing EMD scores for each gene, we use permutations to estimate false discovery rates and obtain *q-*values for each gene. EMD can also be applied to other genomics data, such as copy number values or single cell RNA-Seq data, to identify genes with alteration profiles that are different between two heterogeneous groups. We refer to our method, which is an adaptation of the EMD method to the analysis of Omics data, as *EMDomics.*

We use simulated data as well as real biological data to show the power of EMDomics to identify differentially expressed genes compared to alternative methods when there is both intra- and inter-class heterogeneity. To evaluate the theoretical basis for EMDomics, we used simulated data and compare the performance of EMDomics to SAM and Limma (two of the most commonly used differential expression analysis methods), as well as to Kolmogorov-Smirnov (KS) and Cramer Von Mises (CVM) (two of the most commonly used distribution comparison methods), in terms of sensitivity and specificity for identifying genes truly expressed differentially in the simulation. To evaluate the performance of EMDomics on real biological data, we apply it to the challenging problem of identifying genes associated with drug response in ovarian cancer. Ovarian cancer is the deadliest and the second most common gynecologic cancer (Siegel *et al.*, 2013). Almost all women diagnosed with ovarian cancer receive a combination of cytoreductive surgery and platinum-based chemotherapy (Heintz *et al.*, 2006). Although a subset of patients respond to chemotherapy, the majority do not respond and ultimately succumb to the disease. There have been several published gene expression profiling studies on drug resistance for ovarian cancer, but because of data heterogeneity it has been difficult to identify robust predictors of chemotherapy response using conventional statistical approaches (Konstantinopoulos *et al.*, 2008). We use clinically annotated ovarian cancer gene expression data from The Cancer Genome Atlas (TCGA). We compare EMDomics performance on these data to Limma, SAM and edgeR, as well as to KS and CVM, in terms of power to identify differentially expressed genes, pathways and gene sets associated with drug resistance in ovarian cancer.

## 2 Methods

**Fig. 1.**
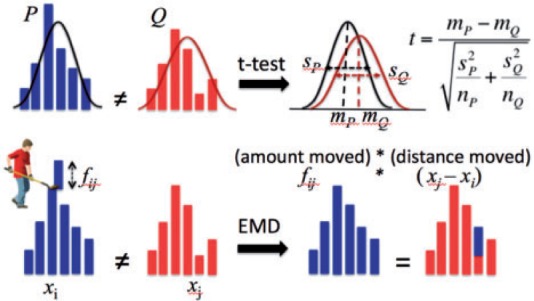
Overview of the EMD method and comparison with a standard t-test for the assessment of differential expression

EMD is a measure of distance between two distributions that reflects the minimum cost of transforming one distribution into the other. If you imagine two piles of dirt (Pile_P_ and Pile_Q_), the EMD_PQ_ is the minimum work required to move dirt from Pile_P_ to Pile_Q_ to make the piles even (Fig. 1). EMD can be computed through solving linear optimization of the classic transportation problem (Dantzig, 1951).

### 2.1 Differential expression analysis using EMD

We developed a new approach (EMDomics) for differential expression analysis of genomics data. Whereas test statistics generated by standard differential expression approaches reflect the likelihood that the difference of mean expression between two groups is non-zero (e.g. SAM and Limma) or reflect the significance of the association between abundance of short reads of the two groups (e.g. edgeR and DEseq), the EMD test statistic reflects the overall difference between two normalized distributions (Fig. 1). The EMDomics method computes the EMD for each gene in a genomewide analysis and determines statistical significance through permutation testing and estimation of false discovery rates (FDRs), indicated by *q-*values (Storey, 2002).

### 2.2 Computing the EMD score

The EMD procedure is more fully described in (Rubner *et al.*, 1998). Briefly, the EMD computes the distance between two distributions, which are represented by signatures. The signatures are sets of weighted features that capture the distributions. For the differential gene expression analysis application, the signatures are data densities computed from gene expression values’ histograms from each class of samples. Two signatures *P* and *Q* can be represented as: *P* = {(*p*_1_, *w_p_*_1_), … , (*p_m_*, *w_p__m_)*}, where *p*_i_ is the center of the *i*_th_ histogram cell and *w_pi_* is the weight of the cell; and *Q* = {(*q*_1_,*w_q_*_1_), … , (*q_n_*,*w*_q_*_n_*)}, where *q_j_* is the center of the *j*_th_ histogram cell and *w_qj_* is the weight of the cell. Given *P*, *Q*, and *d_ij_* (the Euclidean distance between *p_i_* and *q_j_*), the optimization algorithm looks for a flow, *F* = [*f_ij_*] −where *f_ij_* is the flow between *p_i_* and *q_j_* − that minimizes the overall cost (supplementary information):

$$COST(P,Q,\;F)=\overset{m}{\underset{i=1}{\sum }}\overset{n}{\underset{j=1}{\sum }}f_{ij}d_{ij},$$

After finding the optimal flow, *f_ij_*, the EMD is defined as the normalized total cost:

$$EMD\left(P,Q\right)=\frac{\sum_{i=1}^{m}\sum_{j=1}^{n}f_{ij}d_{ij}}{\sum_{i=1}^{m}\sum_{j=1}^{n}f_{ij}}.$$

### 2.3 Calculating *q-*values for EMD scores

The *q-*value is the permutation-based estimate of the FDR when calling significance at that gene’s test statistic level (Storey, 2002). The FDR is the expected proportion of rejected null hypotheses that are rejected incorrectly at a given significance threshold. To calculate the *q-*value of the EMD test, we follow the approach proposed in (Storey, 2002). To generate the null distribution, we permute the sample labels and for each iteration, we compute the EMD between the permuted classes. In our experiments, we performed 1000 iterations, and we used the median of permuted EMDs for each gene as a null distribution to compute FDRs and *q-*values.

To compute the *q-*value for each gene, we obtain FDRs for a range of significance thresholds, from a strict threshold to a lenient one. Given *M* = [*m*_1_, … , *m_N_*], a vector of median of permuted EMDs, and *EMD* = [*emd*_1_, … , *emd_N_*], a vector of observed EMDs, the mathematical representation of FDR for gene *j* and significance threshold *i*, *t_i_*, is as follows:

$$FDR_{ji}=\{\begin{array}{cc} \frac{\sum_{k=1}^{N}I\left(m_{k}>t_{i}\right)}{\sum_{k=1}^{N}I\left(emd_{k}>t_{i}\right)} & if\;emd_{j}\geq t_{i}\\ 1 & otherwise \end{array}.$$

Where *I* is the indicator function, *N* is the number of genes in the dataset, and *t_i_*’s are in descending order from *T* to zero with step Δ: {T, T−Δ, T−2Δ,…,Δ,0} . In our experiments, we set Δ=0.001 and *T* to the rounded maximum EMD minus 1 (*T* = 3). Then, the *q-*value for gene *j* is calculated as:

$$q-{\text{value}}_{j}=\min\left(FDR_{j}\right).$$

## 3 Results

### 3.1 Simulation experiment to evaluate the performance of EMDomics

We designed and performed simulation experiments to evaluate the performance of EMDomics across a variety of models of heterogeneity in a two class problem, ranging from no true intra-class heterogeneity (a single random process within each of the two classes) to more extensive intraclass heterogeneity (a mixture of random processes within one class of samples). Using the simulated datasets, we compared the sensitivity and specificity of EMDomics and SAM for calling significant genes.

**Fig. 2.**
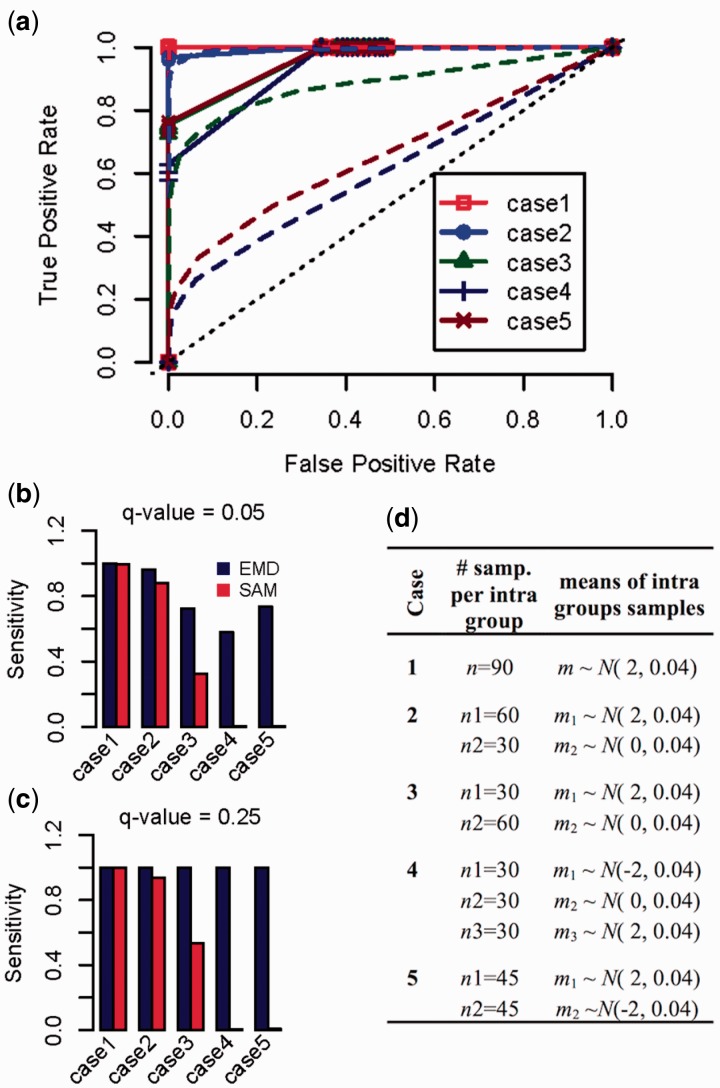
Performance of EMDomics using simulated data. (**a**) ROC curves of EMDomics (solid lines) and SAM (dashed lines) for five cases with different level of heterogeneity in Class 1 samples, ranging from no intra-group heterogeneity (case 1) to significant intra-group heterogeneity (cases 4 and 5) as described in the table (**d**). True positive rates of EMDomics and SAM for (**b**) *q*-value threshold of 0.05 and (**c**) *q-*value threshold of 0.2

The simulated datasets each contain 16 000 genes measured across a total of 240 samples. The samples come from two classes: class 1 contains 90 samples; and class 2 contains 150 samples. Simulated gene expression values for 15 000 genes were drawn from the same Gaussian distribution for the two classes, with zero mean and variance of 1 (*N*(0,1)), which simulate truly non-differentially expressed genes. Gene expression values for the remaining 1000 genes are drawn from different distributions for the two classes and simulate truly differentially expressed genes. For these 1000 truly differentially expressed genes, we considered five cases for the Class 1, ranging from no intra-group heterogeneity to significant intra-group heterogeneity as shown in Figure 2. For all five cases, gene expression values for the Class 1 samples are drawn from Gaussian distributions with variance of 9, but with different means (*m_i_*) (*Class*1 ∼ *N*(*m_i_*, 9)). Gene expression values for Class 2 are drawn from Gaussian distributions with mean (*m*) centered on zero, (*m* ∼ *N*(0, 0.04)), and variance of 9 (*Class*2 ∼ *N*(*m*, 9)).

We applied EMDomics and SAM to the five simulated datasets. We chose SAM, because it calculates *q-*values in the same manner as EMDomics calculates *q-*values, facilitating a fair comparison between the methods. For each simulated case we evaluated a range of *q-*value thresholds for calling significant genes from 0 to 1, and we computed sensitivities (true positive rate) and precisions (1- false-positive rate). The ROC curves for EMDomics and SAM are shown in Figure 2(a). For case one, in which differentially expressed genes are simulated using a single Gaussian distribution with fold change of approximately 2 (no intraclass heterogeneity), EMDomics performs similarly to SAM and both have area under curve (AUC) values close to 1. Adding more heterogeneity to the simulated data resulted in EMDomics outperforming SAM, as indicated by higher true positive rates and lower false positive rates and significantly higher AUC for EMDomics (Fig. 2(a); Table 1). For cases 4 and 5, which represent highly heterogeneous data, EMDomics is still able to detect most differentially expressed genes with a low false positive rate, while SAM performs poorly. Table 1 shows the performance of EMDomics and SAM for a significance threshold of *q-*value = 0.05. Figure 2(b) and (c) show true positive rates of EMDomics and SAM for significance thresholds of *q* = 0.05 and 0.20, respectively. SAM’s sensitivity for identifying significantly differentially expressed genes from highly heterogeneous data (cases 4 and 5) is very low at both significance thresholds, suggesting that EMDomics will increasingly outperform SAM for the identification of differentially expressed genes in the setting of increasing levels of intra-class heterogeneity.

**Table 1.** Area under the curve (AUC), true positive rate (TPR) and false positive rate (FPR) of SAM and EMDomics (TPR and FPR are for *q-*value = 0.05)

Case	AUC		*q* value = 0.05			
			TPR		FPR	
	EMD	SAM	EMD	SAM	EMD	SAM
1	1	0.999	1	0.99	0	1.6 × 10^−3^
2	0.994	0.992	0.96	0.88	0	1.2 × 10^−3^
3	0.957	0.883	0.72	0.32	0	1.3 × 10^−4^
4	0.936	0.611	0.58	0.004	0	0
5	0.962	0.659	0.73	0.005	0	0

To more fully evaluate this hypothesis, we compare the performance of EMDomics with SAM, Limma and edgeR on a real biomedical data set in the following section.

Because the EMD method depends on the comparing the normalized histograms of the two groups, it requires moderate to large sample sizes. We investigated the effects of sample size on specificity and sensitivity of the results using simulated datasets. We generated six simulated datasets with different sample sizes, ranging from 300 to 3 (Supplementary Fig. S1). We generated these datasets for case 1, when there is no intra group heterogeneity, and for case 4, where there is a very high level of intra group heterogeneity. EMDomics results show that there is a sharp decrease in the true positive rate for sample size less than 30 samples in the smallest group for both cases 1 and 4, especially for case 1. Although true positive rates decrease with smaller sample size, false positive rates stay at zero for a wide range of FDR thresholds (FDR threshold < 0.25). Supplementary Figure S1 shows true positive rates and false positive rates for FDR threshold of 0.05 and 0.20.

### 3.2 EMDomics for the identification of genes associated with drug resistance in ovarian cancer

To identify genes associated with drug resistance in ovarian cancer, we applied EMDomics to the processed and normalized ovarian cancer gene expression data from The Cancer Genome Atlas (TCGA). We used gene expression data from the Agilent 244K Custom Gene Expression platform which includes 17 814 genes (Supplementary File 1). As of January 2014, Agilent microarray data of 570 cases with HG-SOC were available in TCGA. Clinical data from these patients were carefully examined to identify eligible samples for assessing cis-platinum chemotherapy response. We used similar definition as used in (Integrated genomic analyses of ovarian carcinoma, 2011) for resistant and sensitive tumors. Tumors were defined as sensitive if after the last primary treatment the platinum free interval was 6 months or greater, there was no evidence of progression or recurrence, and the follow-up interval was at least 6 months from the date of last primary platinum treatment. Tumors were defined as resistant if the patient recurred within 6 months after the last treatment (Integrated genomic analyses of ovarian carcinoma, 2011). Among the 570 cases, we identified 331 cases with explicit cis-platinum status, with 97 platinum resistant and 234 platinum sensitive primary tumors. The clinical information of the resistant and sensitive samples is given in the Supplementary Table S1.

**Fig. 3.**
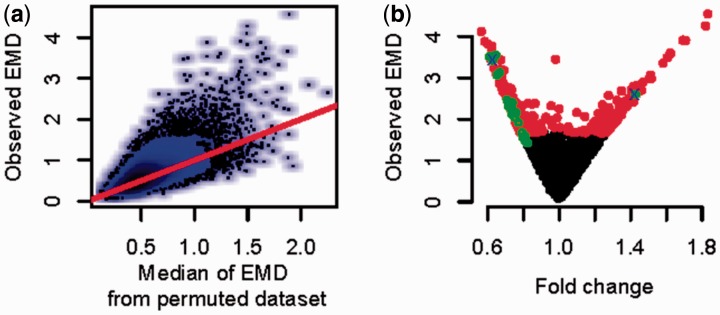
Performance of EMDomics for identifying genes associated with drug resistance in ovarian cancer. (**a**) Scatter plot of the median of permuted EMD values versus observed EMD values for the TCGA dataset. Black dots represent genes. The red line has a slope of 1 and passes through the origin. (**b**) EMD score versus fold change for TCGA dataset; red dots are significant genes with *q-*value < 0.05 using EMDomics, green circles are significant genes with *q-*value < 0.05 using SAM, and blue crosses are significant genes with adjusted *p*-value < 0.05 using Limma

Figure 3(a) shows the scatter plot of observed EMD scores versus median of permuted EMD scores. Since EMD is a measure of the distance between two distributions it is a positive number. As expected, observed EMD scores tend to be larger than the median of permuted EMD scores. Histograms of the permuted EMD values and observed EMD values are shown in Supplementary Figure S2.

In differential expression analysis, the fold change (the ratio of the mean (or median) expression level of a gene between two classes) is commonly used as a metric to indicate the magnitude of expression change across two classes and is a natural complement to the *t*-test, which is a measure of the statistical significance of differences in mean expression between two classes. However, in the setting of significant intraclass heterogeneity, fold change may not be a suitable indicator of the magnitude of differential expression between groups, and EMD may be a more useful metric. Figure 3(b) is a scatter plot of EMD versus fold change for all genes and shows that fold change and EMD are only moderately correlated, and a significant proportion of genes have low fold change (close to one) but large EMD (two and more), which supports the hypothesis that genes can show significant differences in overall expression distribution between classes, while at the same time showing little difference in mean expression between classes.

We also applied EMDomics to the TCGA ovarian cancer RNA-Seq data, which is available for a subset of ovarian cancer samples in TCGA. This dataset contains 30 resistant and 67 sensitive samples. After filtering out genes that in more than 50% of samples had a normalized read count less than 20, the data set includes 8106 genes. We applied EMDomics to the log 2 normalized read count data (transcript per million (TPM)) for genes after quantile normalization. We observed similar behavior in the EMDomics results compared to those obtained when applying EMDomics to array-based data. The scatter plot of observed EMD scores versus median of permuted EMD scores and scatter plot of EMD versus fold change for all genes are shown in Supplementary Figures S3 and S4. We also compared the EMDomics results of the set of genes measured across the two platforms. These data show that although there are differences across microarray and RNAseq, overall, we see significant correlation of the EMDomics measures across the two platforms (Supplementary Fig. S5).

### 3.3 Comparison of EMDomics with conventional differential expression analysis methods.

We compared the performance of EMDomics with that of SAM, Limma and edgeR for the identification of genes associated with drug response in ovarian cancer. We applied SAM and Limma to the processed and normalized TCGA array-based gene expression dataset using the samr (Tibshirani *et al.*, 2011) and Limma (Smyth, 2005) R packages from Bioconductor (Gentleman *et al.*, 2004). We also applied edgeR to the RNA-Seq data of the subset of samples with RNA-Seq data and genes that passed filtering (as described above) using the edgeR (Robinson *et al.*, 2010) package from Bioconductor.

Using the array-based gene-expression data, while the *q-*values and adjusted *p*-values of SAM and Limma are highly correlated (R = 0.98, Supplementary Fig. S6) with each other, the *q-*values and adjusted *p*-values of SAM and Limma are only moderately correlated with the *q-*values of EMD (*R* = 0.59 and 0.53, respectively, Supplementary Fig. S7). Overall, EMDomics identifies far more statistically significant genes associated with drug resistance (475 genes with *q* < 0.05), as compared with SAM (23 genes) or Limma (2 genes) (lists of all genes with their EMD, SAM and Limma significant values are in Supplementary File 2). While EMD identifies significant genes across a wide range of fold-change values, significant genes identified by SAM and Limma are limited to genes showing at least moderate fold change in mean expression between classes (Fig. 3(b); Supplementary Fig. S8).

**Fig. 4.**
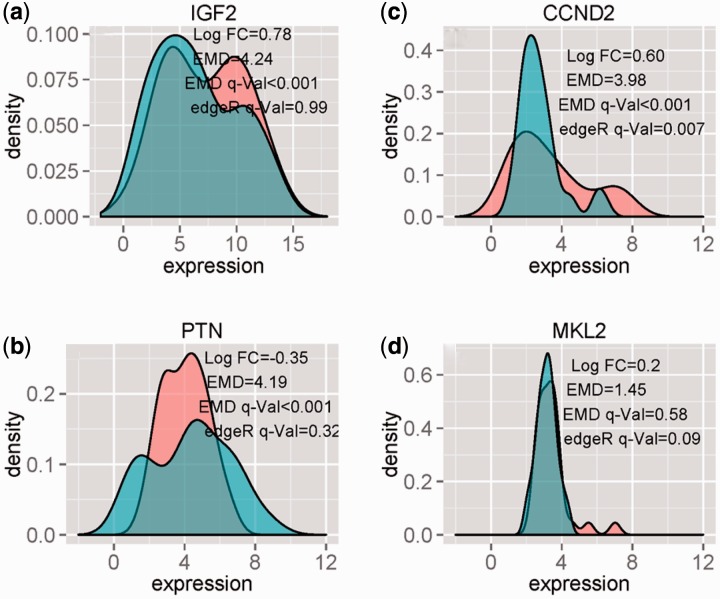
Density plots of IGF2, PTN, CCND2 and MKL2 for resistant (red) and sensitive (blue) samples using normalized read count data (TPM) from TCGA RNA-Seq data

EMDomics shows similar performance when using RNA-Seq based expression values (normalized read counts). However because of higher level of variability among read count data, less number of samples and shorter gene list, EMDomics identifies fewer significant genes (30 genes with *q* < 0.05) than were identified using the array-based data. However, edgeR identified only one gene as significant with *q* < 0.05 (CCND2) on the RNA-Seq data. CCND2 was also identified as significant by EMDomics. The table including EMDomics and edgeR results on the RNA-Seq data for the 8106 genes is provided in Supplementary File 3. The density plots of CCND2 (which is called significant by EMDomics and edgeR), IGF2 and PTN (which are called significant by EMDomics but not by edgeR) and MKL2 (with edgeR *q-*value = 0.09, as compared with EMDomics *q* value = 0.58) is shown in Figure 4. While edgeR is more sensitive to the genes that have a narrow read count distribution with a long tail (one or two samples have significantly more read count compare to the rest of samples (Fig. 4(d)), EMDomics identifies genes with read count distributions that are wide (high intra-group heterogeneity) and significantly variable between groups (Fig. 4(b) and (c)).

**Fig. 5.**
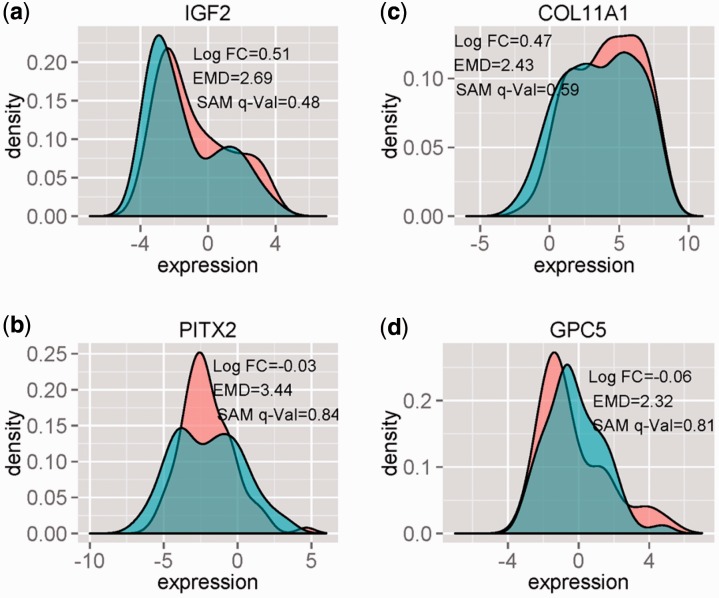
Density plots of GPC5, PITX2, IGF2 and COL11A1 for resistant (red) and sensitive (blue) samples, using array-based expression value data from TCGA

Since more samples and genes are available from the array-based data as compared with the RNA-Seq data, the array-based data was used for the functional gene set analyses. The 15 genes with the lowest *q-*value in EMDomics (EMDomics *q* < 0.001) and highest *q-*values in SAM (SAM *q* > 0.47) are listed in Supplementary Table S2. All of these genes, but one, have been previously associated with cancer (Alanen *et al.*, 2000; Basu and Roy, 2013; Brouwer-Visser *et al.*, 2014; Fung *et al.*, 2012; Huang *et al.*, 2010; Kuhn *et al.*, 2012; Moon *et al.*, 2003; Ohmachi *et al.*, 2006; Pearce *et al.*, 2011; Teng *et al.*, 2014; Wu *et al.*, 2014; Yang *et al.*, 2014) and several have been specifically implicated in ovarian cancer progression. For example, GPC5 is associated with many cancers, including ovarian cancer (Integrated genomic analyses of ovarian carcinoma., 2011), suggesting its relevance to this biomedical problem; PITX2 has been associated with ovarian cancer progression and malignant phenotypes in both observational (Fung *et al.*, 2012) and mechanistic (Basu and Roy, 2013) studies; the IGF signaling pathway is associated with drug resistance in several cancers (Brouwer-Visser *et al.*, 2014; Huang *et al.*, 2010; Pearce *et al.*, 2011); and COL11A1 is associated with poor outcome and resistance to cis-platinum in ovarian cancer cell lines (Teng *et al.*, 2014; Wu *et al.*, 2014). Figure 5 shows the density plots of GPC5, PITX2, IGF and COL11A1 for resistant and sensitive samples−the distributions of gene expression values are wide within both the sensitive and resistant classes (high intraclass heterogeneity), and there are clear differences in the shapes of distributions between the sensitive and resistant groups.

23 genes are called significant by SAM with *q-*value < 0.05 (T S2). Each of these genes but four (TACSTD1, MSH6, SPCS2, ZNF592) are called significant by EMDomics, as well. Supplementary Figure S9 shows the density plots of these four genes for resistant and sensitive samples. Among them, TACSTD1 and MSH6 have been associated with cancer in the literature. For these genes, the distributions of gene expression values in sensitive and resistant samples are very narrow (low variance), which drives the high test statistic and low *q-*value from the *t-test* in Limma and SAM. MATN2 and LCTL were the only two genes called significant by Limma at an adjusted *p*-value < 0.05 (Supplementary Table S3). These two genes were both called significant by EMDomics, as well. The density plots of four top genes (SERTAD4, LIPG, MATN2, GJB1) called significant by both SAM and EMDomics are shown in Supplementary Figure S10. These genes show differences in both mean expression levels and distribution shape between sensitive and resistant samples.

In summary, EMDomics identifies far more differentially expressed genes than SAM or Limma, and the genes prioritized by EMDomics appear to be highly relevant for ovarian cancer biology.

To compare the robustness of differential expression results between EMDomics and SAM, we performed bootstrapping analyses using both approaches. Across 100 bootstrap iterations and a significance threshold of FDR = 5% applied at each iteration, we identify a median of 302 genes (95% confidence interval 171–583) differentially expressed by EMDomics compared with a median of only five genes (95% confidence interval 0–45) by SAM. These results support the robustness of the observation that EMDomics is a more powerful method than SAM for the identification of drug resistance associated genes in ovarian cancer. Next, we used bootstrapping to compare the ability of EMDomics and SAM to identify genes that repeatedly attain statistical significance across the bootstrap iterations. Across the 100 bootstrap iterations, we identify 125 genes with a median *q-*value < 5% with EMDomics, and we identify no genes with a median *q-*value < 5% using SAM. These results show that EMDomics significantly outperforms SAM for the identification of genes with robust association with drug resistance in ovarian cancer.

To compare the biological cohesiveness of genes prioritized by EMDomics and SAM, we performed a comparative gene set enrichment analyses on sets of top significant genes identified by the two methods.

### 3.4 Enrichment analysis

#### 3.4.1 Gene Set Enrichment Analysis (GSEA)

We used the “Investigate Gene Sets” function of the web-based GSEA tool provided by the Broad Institute (http://www.broadinstitute.org/gsea/msigdb/annotate.jsp) to assess overlap of genes called significant by EMDomics and SAM (*q-*value < 0.05) and gene sets in the Canonical Pathways (CP), KEGG database (CP:KEGG), and oncogenic signatures (C6) in the Broad Institute’s Molecular Signatures Database (MSigDB) (Total number of considered gene sets = 1 509; 1320 gene sets for CP and CP:KEGG, and 189 gene sets for C6). Using EMDomics, we identify 99 C6 gene sets and 10 CP and CP:KEGG gene sets, with *q-*value for enrichment < 0.01. Using SAM significant gene lists, we identify no significantly enriched gene sets.

The top 10 most significantly enriched gene sets identified by EMDomics are listed in Supplementary Table S4. This list of most highly enriched gene sets includes pathways known to play critical roles in ovarian cancer pathogenesis. For example, TP53 mutations occur in almost all high grade serous ovarian cancers (Integrated genomic analyses of ovarian carcinoma., 2011) and altered TP53 function is known to play a central role in drug resistance in ovarian cancer (Shelling, 1997). The most enriched gene set identified by the EMDomics analysis is a set of genes down-regulated in cancer cell lines with mutated TP53 (*q*-value = 9.33e − 14), suggesting the value of EMDomics for prioritizing pathways highly relevant to drug response in ovarian cancer. Other gene sets identified as highly enriched by the EMDomics analysis include gene sets related to: LEF1, BMI1, KRAS, EZH2, and PTEN, and pathways related to cell-cell junction organization, cell-cell communication, WNT signaling, and extracellular matrix organization, each of which have been previously implicated in cancer progression.

**Fig. 6.**
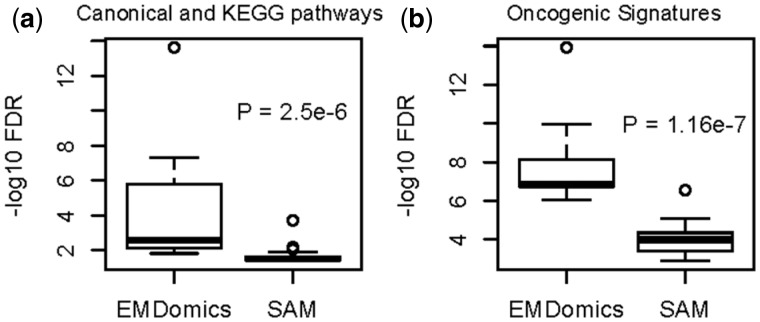
Box plots of FDRs of enrichment analysis of top-ranked genes identified by EMDomics and SAM, using the ‘Investigate Gene Sets’ function of the web-based GSEA tool. (**a**) *q-*values for the top 20 enriched Canonical and KEGG pathways in the 475 top-ranked genes. (**b**) *q-*values for the top 20 enriched oncogenic signatures in the 475 top-ranked genes

Next, we repeated the comparative gene set enrichment analysis using the exact same number of top-ranked input genes (*n* = 475 genes) for EMDomics and SAM. With *q*-value < 0.05, the set of 475 significant genes identified by EMDomics is enriched for 31 Canonical and KEGG Pathways, while the set of 475 top-ranked genes identified by SAM is only enriched for 21 Canonical and KEGG Pathways. We also assessed the statistical strength of the gene set enrichments for genes identified by EMDomics as compared with those identified by SAM. Considering the top 20 ranked pathways, EMDomics identifies significantly stronger enrichments than SAM (Wilcoxon test *P* = 2.5 × 10^−^^6^, Fig. 6(a)). We observed similar behavior for the oncogenic signatures enrichment analysis. 99 oncogenic signatures gene sets are enriched at (*q*-value < 0.05) in the 475 top-ranked EMDomics genes, while only 39 oncogenic signatures gene sets are enriched at this significance threshold in the 475 top-ranked SAM genes. Considering the top 20 significant oncogenic signatures, EMDomics again identifies significantly stronger enrichments (Wilcoxon test *P* = 1.16 × 10^−^^7^, Fig. 6(b)). The Box plots of FDRs of enrichment analyses on genes identified as top-ranked by only SAM or by only EMDomics, but not by both are provided in the Supplementary Figure S11. These data show that EMDomics outperforms SAM for the prioritization of genes enriched for oncogenic signatures and biological pathways. The top 10 oncogenic signatures and pathways enriched in: the 475 top-ranked genes by SAM; the unique genes among the top-ranked EMDomics genes; and in the unique genes among the top-ranked SAM genes are shown in Supplementary Tables S5–S8, respectively.

#### 3.4.2 Gene ontology processes, diseases by biomarkers and pathway maps enrichment analysis

**Fig. 7.**
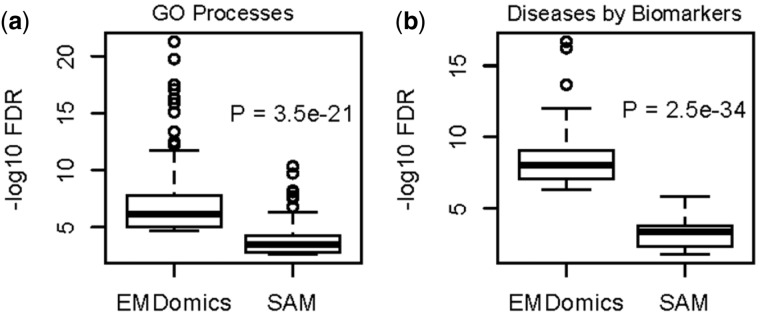
Box plots of FDRs of enrichment analysis of top-ranked genes identified by EMDomics and SAM, using the MetaCore web-based enrichment analysis tool. (**a**) *q* values for the top 100 enriched GO Processes in the 475 top-ranked genes. (**b**) *q*-value for the top 100 enriched Diseases by Biomarkers in the 475 top-ranked genes

We used the MetaCore (http://thomsonreuters.com/metacore/) software tool to perform enrichment analyses of: Gene Ontology (GO) Biological Processes, Diseases by Biomarkers, and Pathway Maps using the list of EMDomics significant genes (475 genes) and the list of 475 top-ranked SAM genes. Similar to gene set enrichment analysis described above, more GO Processes and Diseases by Biomarkers (1368 GO Processes and 551 Diseases) are enriched in EMDomics significant genes (with *q*-value < 0.05) compared to those enriched in the 475 top-ranked genes by SAM (603 GO Processes and 136 Diseases). Considering the top 100 enriched GO Processes and Diseases, we observed that EMDomics identifies significantly stronger enrichments (Wilcoxon *P* < 2 × 10^−^^20^ for GO Processes and Diseases by Biomarkers) compared to SAM (Fig. 7(a) and (b)). The gene set enrichment analyses on genes identified as top-ranked by only SAM or by only EMDomics, but not by both are given in the Supplementary File 4 and Figures S12, S13, and S14.

Pathway Maps enrichment analysis of the EMDomics significant genes yielded six enriched maps (with *q*-value < 0.05), which includes: Angiotensin system maturation, WNT signaling pathway, Extracellular matrix (ECM) remodeling, and PGE2 pathways in cancer, which are all implicated in ovarian cancer progression (Arend *et al.*, 2013; Herr *et al.*, 2013; Januchowski *et al.*, 2014; Polakis, 2000; Rask *et al.*, 2006). The only map enriched by SAM’s top-ranked genes (*q*-value < 0.05) is Cadherin-mediated cell adhesion. A list of the significant enriched Pathway Maps from the EMDomics significant genes is given in Supplementary Table S9).

Enrichment analysis of the top-ranked differentially expressed genes, identified by EMDomics and by SAM, shows that EMDomics has more power to identify biological gene sets and pathways differentially expressed between two heterogeneous groups.

### 3.5 Comparison of EMDomics with conventional distribution comparison methods

The Kolmogorov–Smirnov (KS) and Cramer Von Mises (CVM) tests, which quantify the distance between two distributions, are common nonparametric tests to compare two groups of samples. In KS the test metric is the maximum distance between the two cumulative distribution functions (CDFs); and it is invariant to arbitrary monotonic differences between the two distributions. The KS test differs from EMDomics, since EMD considers all the differences (incorporating both quantity and distance of differences) between two distributions, while KS measures only the maximum difference between two CDFs. As expected, EMDomics shows more power compared to KS to capture intra-class heterogeneity on both simulated and real biological data. In simulated data KS performs similarly to EMDomics when there is no intra-class heterogeneity (case 1 in Fig. 2). The AUC for KS in case 1 is 0.98. By increasing the intra-class heterogeneity, KS fails to identify truly differentially expressed genes. Its true positive rates for case 4 and 5 are 0. Supplementary Figure S15 shows ROC curves for KS compared to EMDomics for the five cases in Figure 2. Table 2 shows the AUCs, false positive rates (FPRs) and true positive rates (TPRs) for a significance threshold of *q*-value = 0.05. We applied KS to the ovarian cancer expression data for resistant and sensitive groups. KS identifies no significantly differentially expressed genes with adjusted *p*-value < 0.05. Supplementary Figure S16 shows KS adjusted *p*-values versus EMDomics *q-*values.

**Table 2.** Area under the curve (AUC), true positive rate (TPR) and false positive rate (FPR) of EMDomics, CVM and KS (TPR and FPR are for *q*-value = 0.05).

Case	AUC			*q*-value = 0.05					
				TPR			FPR		
	EMD	CVM	KS	EMD	CVM	KS	EMD	CVM	KS
**1**	1	0.98	0.98	0.99	0.99	0.95	0	0.04	0.02
**2**	0.99	0.96	0.94	0.92	0.92	0.60	0	0.04	0.01
**3**	0.95	0.92	0.74	0.71	0.73	0.04	0	0.04	0
**4**	0.93	0.88	0.57	0.51	0.55	0	0	0.04	0
**5**	0.94	0.89	0.64	0.56	0.58	0	0	0.03	0

In CVM, the test metric is the sum of the squared values of difference between two CDFs. CVM is a special case of EMD; it is one of the solutions for making two distributions even, but it is not guaranteed to be the optimal solution. Because it adds all the squared values of differences between two CDFs, CVM tends to overestimate the mutual similarity and it is not able to effectively handle partial matches. To explore the CVM performance, we applied it to the simulated and the real biological data. To compute *q-*values for CVM, we used the same approach that we employed for calculating *q-*values for EMDomics. Using simulated data, CVM shows comparable performance to EMDomics. The TPRs of CVM are very close to those of EMDomics, however CVM generates more false positives. While the FPRs of EMDomics are almost zero, CVM has FPRs of about 4% (Table 2). This difference in FPRs results in slightly lower AUC for CVM compared to EMDomics, as shown in Table 2 and Figure S14. We also applied CVM to the ovarian cancer gene expression data. CVM identified far more significant genes (2991 genes) compared to EMDomics (475 genes) with *q-*value < 0.05. All genes identified as significant by EMDomics are called as significant by CVM as well. Thus, CVM and EMDomics show similar overall performance on calling true positives on the simulated data; however, given EMDomics’s lower rate of false positives on the simulated data, EMDomics may represent a more precise and conservative approach than CVM. The result of applying EMDomics, KS and CVM on the array-based TCGA ovarian cancer is given in Supplementary File 5.

## 4 Discussion

In this work we proposed to use the distance between the distributions of expression values (for array-based data) or normalized read counts (for sequence-based data) to identify differentially expressed genes when there is a high level of heterogeneity between and within the groups. We developed a new method (EMDomics), based on the Earth Mover's Distance (EMD), for computing the distance between the distributions of expression values or normalized read counts and for the identification of genes significantly differentially expressed between heterogeneous groups.

Conventional methods, such as *t*-test-based or Fisher exact test-based approaches, perform well for the identification of genes differentially expressed between homogeneous classes of samples. However, many problems in biology and biomedicine contain samples that show both significant intra- and inter-class heterogeneity. If the sources and structure of the heterogeneity are known ahead of time, then they may be incorporated into the analysis using conventional approaches (e.g. stratification, inclusion of an interaction term); however, frequently the sources and structure of intra-group heterogeneity are unknown. In these cases, failure to account for the presence of intra-group heterogeneity will result in high intra-group variance driving high *p*-values, even for genes that may truly be associated with a biological class.

Our analyses, on both simulated and real data, show that considering whole distributions with EMDomics has more power to capture heterogeneity and identify genes and gene sets that are expressed differently between two heterogeneous groups.

The EMDomics method has several strengths and limitations. Its primary strength is that it is a robust non-parametric method, which does not make any assumptions about the distributions or differences between the two classes being compared, and thus has significantly more power than conventional approaches for identifying differential Omics features between heterogeneous classes. Thus, the method can be applied in a wide variety of settings to compare distributions of Omics data between two classes. A further strength of the method is that efficient algorithms are available to compute EMD (Rubner *et al.*, 2000).

However, ‘there is no free lunch in statistics’ (Simon and Tibshirani, 2014; Wolpert and Macready, 1997), and EMDomics has a few limitations. First, EMDomics can currently only be used for two class problems and cannot be used with quantitative or multiclass labels. Second, because EMD is based on comparing the histograms of the two groups it requires at least a moderate sample size (∼30 samples per class) and will tend to perform poorly when there are few samples. Third, a significant EMD value does not allow any inference to be made regarding the direction or structure of an association; it provides evidence that a gene is expressed differently (in some way) between two classes, which can then lead to further follow-up analyses and experimental studies to more precisely characterize the association and its clinical and biological significance.

Despite the method’s limitations, our data suggest that EMDomics is a powerful new approach for identifying differentially expressed genes between heterogeneous classes of samples. Although we demonstrate one application of EMDomics, it can apply to identify other genomics data types (such as copy number variation data), as well as to other types of populations (such as populations of single cell measurements). Given ongoing major efforts to generate massive Omics datasets (ranging in scale from single cell genomics to population-based Omics studies in molecular epidemiology) to investigate a wide array of questions in bio-medicine, we expect EMDomics to be a useful new tool for the identification of genes and gene sets differentially expressed between heterogeneous classes of samples.

## Supplementary Material

Supplementary Data
