# Supplementary material for: EMDomics: a robust and powerful method for the identification of genes differentially expressed between heterogeneous classes
Source: Bioinformatics. 2015 Oct 29;32(4):533–41. doi: 10.1093/bioinformatics/btv634 (PMC4743632; doi:10.1093/bioinformatics/btv634)
Supplement: Supplementary Data [file supp_32_4_533__index.html]

EMDomics: a robust and powerful method for the identification of genes differentially expressed between heterogeneous classes — EMDomics: a robust and powerful method for the identification of genes differentially expressed between heterogeneous classes — Supplementary Data 

# EMDomics: a robust and powerful method for the identification of genes differentially expressed between heterogeneous classes

## Supplementary Data

files

- Supplementary Data - zip file
