# Supplementary material for: EMDomics: a robust and powerful method for the identification of genes differentially expressed between heterogeneous classes
Source: Bioinformatics. 2015 Oct 29;32(4):533–41. doi: 10.1093/bioinformatics/btv634 (PMC4743632; doi:10.1093/bioinformatics/btv634)
Supplement: Supplementary Data [file supp_btv634_suppl_data.zip › SuplementaryInfromation_revised_clean.pdf]

Supplement to "EMDomics: a robust and powerful method for the identification of genes differentially expressed between heterogeneous classes"

Computing EMD

The EMD computes the distance between two distributions, which are represented by signatures. The signatures are sets of weighted features that capture the distributions. For the differential gene expression analysis application, the signatures are data densities computed from gene expression values' histograms from each class of samples. Two signatures P and Q can be represented as: $P = \{(p_1, w_{p_1}), \ldots, (p_m, w_{p_m})\}$, where p_i is the center of the i_{th} histogram cell and w_{p_i} is the weight of the cell; and $Q=\{(q_1,w_{q_1}),\ldots,(q_n,w_{q_n})\}$, where q_j is the center of the j_{th} histogram cell and w_{q_j} is the weight of the cell. Given P, Q, and d_{ij} (the Euclidean distance between p_i and q_j), the optimization algorithm looks for a flow, $F=[f_{ij}]$ −where f_{ij} is the flow between p_i and q_j − that minimizes the overall cost (supplementary information):

$$\mathrm{COST}(P,Q,F) = \sum_{i=1}^{m}\sum_{j=1}^{n} f_{ij} d_{ij} \quad ,$$

subject to the following constraints:

$$f_{ij} \geq 0, \qquad 1 \leq i \leq m,\ 1 \leq j \leq n\ ,$$

$$\sum_{j=1}^{n} f_{ij} \leq w_{p_i} \qquad 1 \leq i \leq m\ ,$$

$$\sum_{i=1}^{m} f_{ij} \leq w_{q_j} \qquad 1 \leq j \leq n\,,$$

$$\sum_{i=1}^{m}\sum_{j=1}^{n} f_{ij} = \min\left(\sum_{i=1}^{m} w_{p_i}, \sum_{j=1}^{n} w_{q_j}\right).$$

The above constraints allow only transferring earth from P to Q , limit the amount of earth that can be sent by the cells in P to their weights, limit the cells in Q to receive no more earth than their weights, and forces movement of the maximum amount of earth given the other constraints.

After finding the optimal flow, f_{ij} , the EMD is defind as the normalized total cost:

$$\mathrm{EMD}(P,Q) = \frac{\sum_{i=1}^{m}\sum_{j=1}^{n} f_{ij}\, d_{ij}}{\sum_{i=1}^{m}\sum_{j=1}^{n} f_{ij}}.$$

Enrichment analysis of top-ranked genes by only SAM and by only EMDomics

We also focused our enrichment analyses on genes identified as top-ranked by only SAM or by only EMDomics, but not by both. Of the total of 475 top-ranked genes identified by EMDomics and by SAM, 182 (38%) genes were identified by both EMDomics and SAM (common genes between EMDomics significant genes and the 475 top-ranked genes identified by SAM), and 293 genes were identified by SAM only and by EMDomics only. 25 Canonical and KEGG Pathways gene sets and 94 oncogenic signatures are enriched by only EMDomics significant genes (293 genes that are not among SAM 475 top-ranked genes), compared with only 14 Canonical and KEGG Pathway gene sets and 19 oncogenic signatures enriched by only SAM top-ranked genes (293 genes that are not among EMDomics significant genes), demonstrating that the EMDomics-only gene list is enriched for significantly more gene sets as compared with the SAM-only gene list. Applying Wilcoxon test on the top 14 enriched Canonical and KEGG Pathways, as well as on the top 19 oncogenic signatures shows the ability of EMDomics to identify stronger gene set enrichments ($P=6.7\times10^{-6}$ for the Canonical and KEGG Pathways enrichment analysis and $P=1.16\times10^{-7}$ for the oncogenic signatures enrichment analysis, Figs S9(a) and S9(b)). Thus, compared with SAM, EMDomics identifies more enriched gene sets with stronger levels of enrichment.

We also used genes that are only in the EMDomics significant genes (EMDomics-only) and are only in the SAM 475 top-ranked genes (SAM-only) for GO Biological Processes and Diseases by Biomarkers enrichment analysis. More GO Biological Processes and Diseases by Biomarkers, with higher confidence, are enriched in the EMDomics-only significant genes (genes that are not among the top-ranked SAM's genes) compared to those enriched in only top-ranked SAM's genes (genes that are not among EMDomics significant genes). Specifically, 1,263 GO Processes and 576 Diseases (by Biomarker) are enriched among the EMDomics-only significant genes; while only 603 GO Processes and 17 Diseases by Biomarkers are enriched in SAM-only top-ranked genes. Using the top 100 enriched GO Processes and the top 17 enriched Diseases, we observed that EMDomics identifies significantly stronger enrichments (Wilcoxon $P=1.8\times10^{-26}$ for GO Processes and $P=6.8\times10^{-7}$ for Diseases by Biomarkers, Figs S10(a) and S10(b)). Interestingly, the top 10 enriched diseases by the EMDomics-only significant genes are all cancers. Figs. S11 and S12 show the enriched GO Processes and Diseases by Biomarkers when EMDomics-only significant genes, SAM-only, and common genes between SAM's top-ranked genes and EMD's significant genes. The details of the enriched GO Processes and Diseases by Biomarkers are provided in Supplementary File S4.

Supplementary Figures

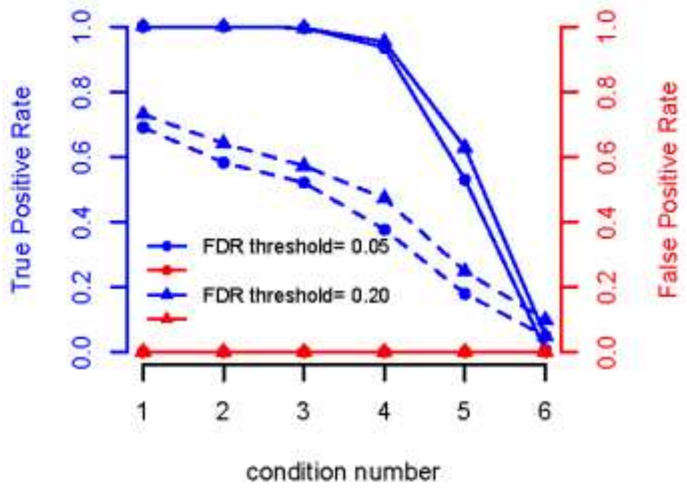

Condition	nRes # samples in group 1	nSen # samples in group 2
**1**	180	300
**2**	90	150
**3**	60	100
**4**	30	50
**5**	10	10
**6**	3	3

Fig. S1. True positive rates (blue lines) and false positive rates (red lines) for FDR threshold of 0.05 (circle markers) and 0.20 (triangle marker) when there is no intra group heterogeneity (Case1, solid line) and when there is a significant level of heterogeneity (Case4, dashed line)

(a)

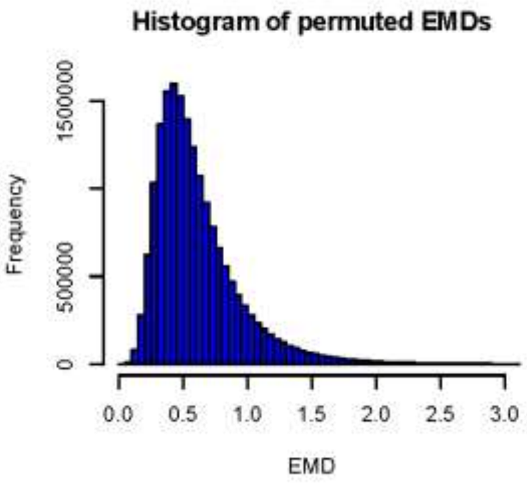

(b)

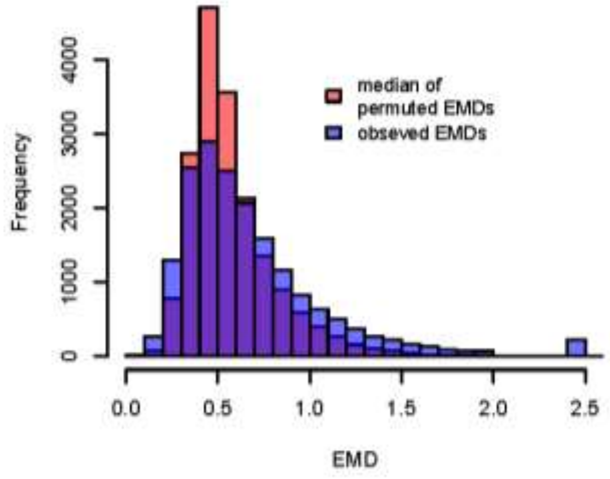

Fig. S2. (a) Histogram of permuted EMD values, and (b) histograms of medians of permuted EMDs (red bars) and observed EMDs (blue bars) for each gene, EMDs greater than 2.5 are set to 2.5.

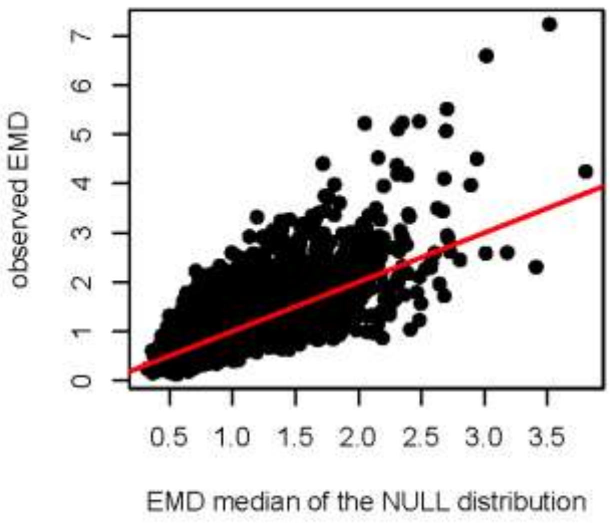

Fig. S3. Scatter plot of the median of permuted EMD values vs. observed EMD values for the RNA-seq TCGA dataset. Black dots represent genes. The red line has a slope of 1 and passes through the origin.

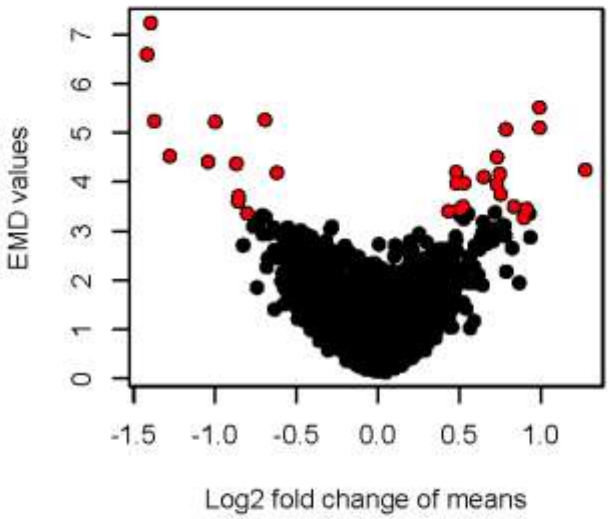

Fig. **S4.** EMD score vs fold change for RNA-seq TCGA dataset; red dots are significant genes with q-value < 0.05 using EMDomics.

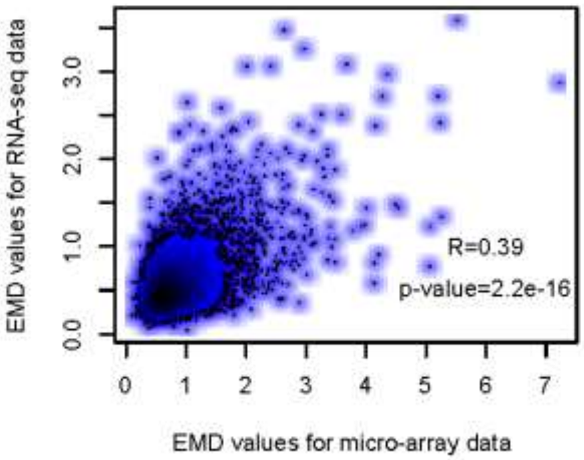

Fig. **S5.** EMD score from microarray data vs EMD score from RNA-seq data.

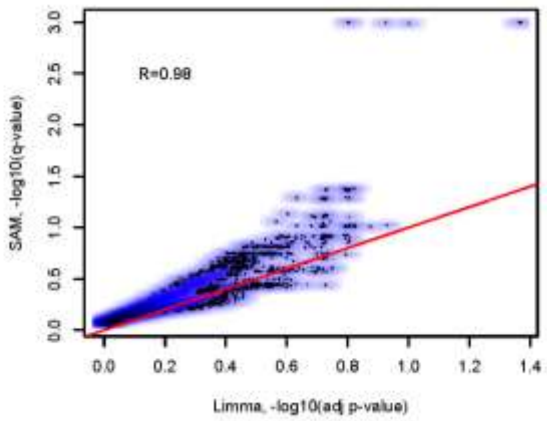

Fig. S6. q-value of SAM versus adjusted p-value of Limma. As expected SAM's q-values and Limma's adjusted p-values are highly correlated. q-values of zeros are set to 10^{-3}.

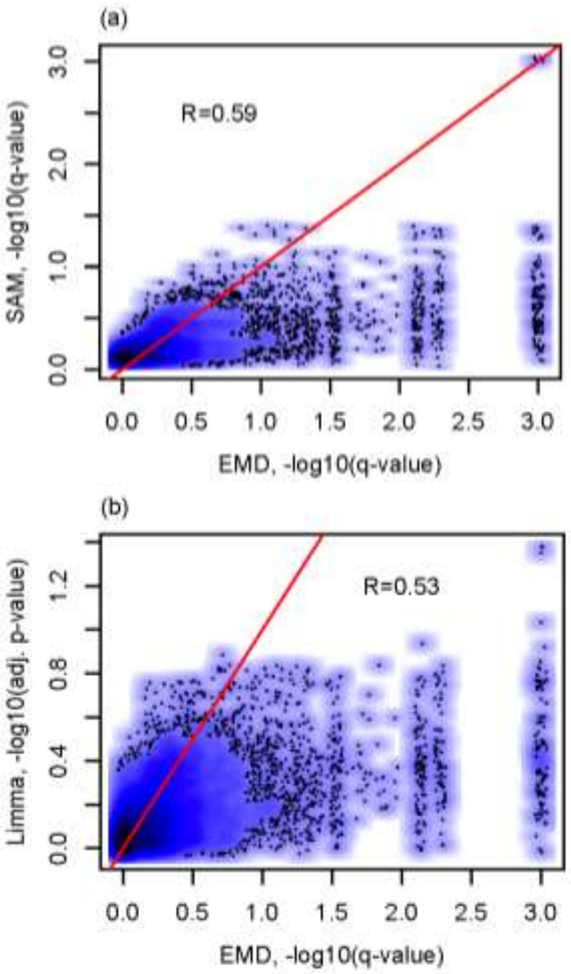

Fig. S7. Comparison of EMDomics' q-values to SAM's and Limma's q-values. (a) q-value of EMDomics versus q-value of SAM. (b) q-value of EMDomics versus adjusted p-value of Limma. q-values of zeros are set to 10-3. The red line has a slope of 1 and passes through the origin.

(a)

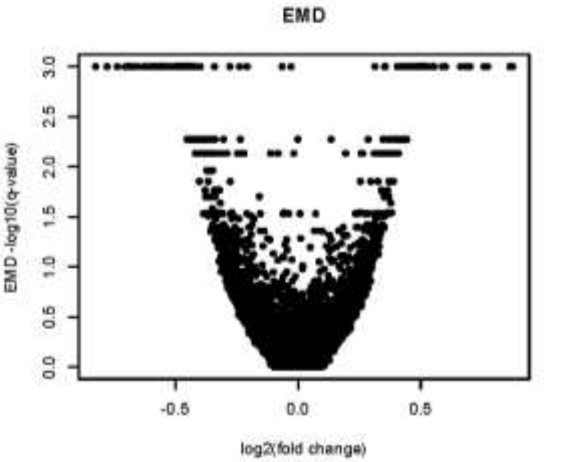

(b)

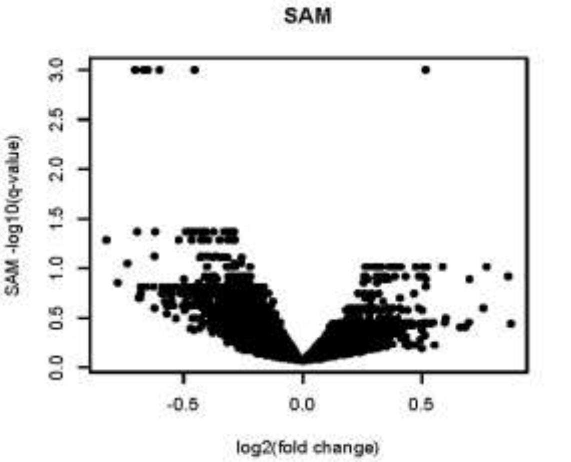

(c)

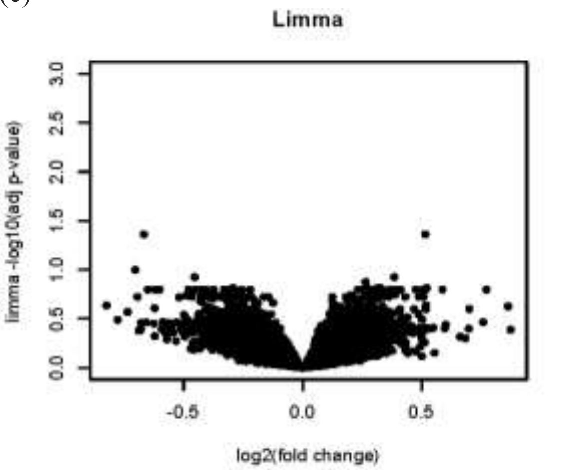

Fig. S8. (a) q-value of EMDomics versus log2 fold change of mean expression, (b) q-value of SAM versus log2 fold change of mean expression, and (c) adjusted p-value of Limma versus log2 fold change of mean expression. zeros are set to 10^{-3}.

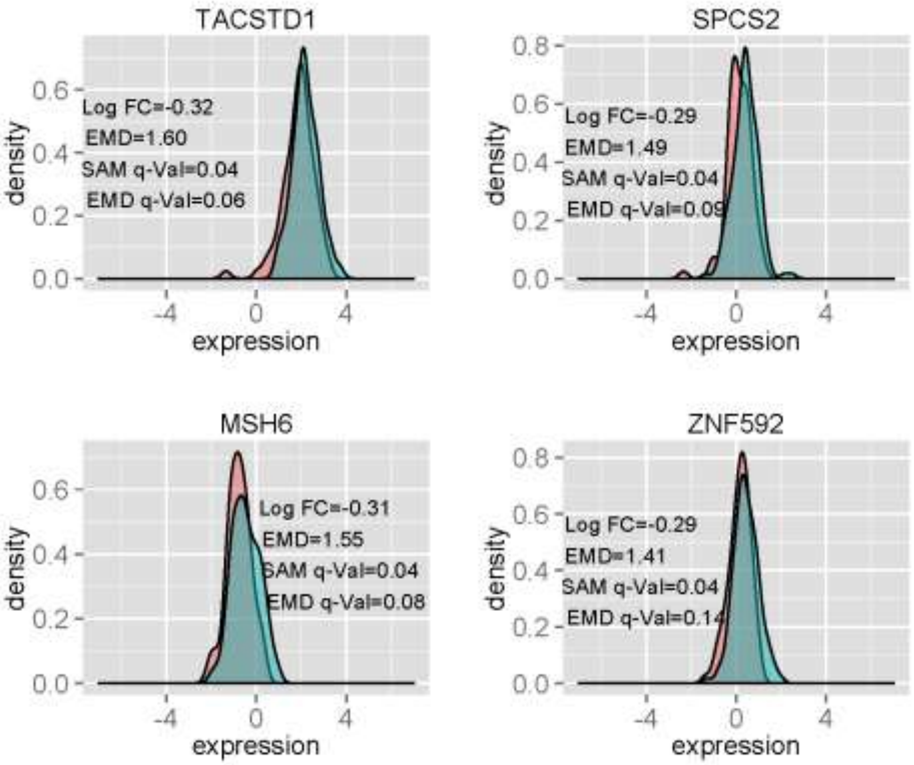

Fig. **S9.** Density plots of TACSTD1, MSH6, SPCS2, and ZNF592 that are called as significant by SAM but not by EMDomics, for resistant (red) and sensitive (blue) samples.

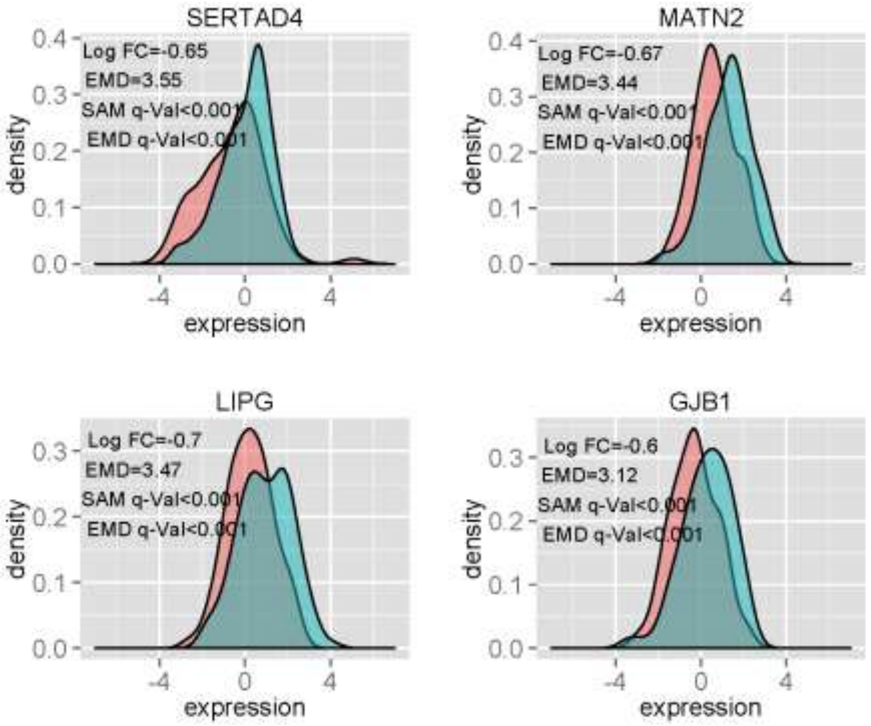

Fig. **S10.** Density plots of SERTAD4, LIPG, MATN2, and GJB1 that are called significant by both SAM and EMDomics, for resistant (red) and sensitive (blue) samples.

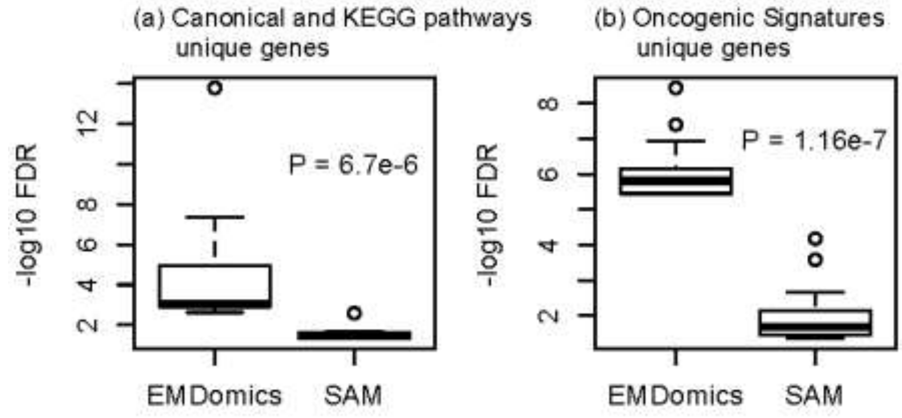

Fig. S11. Box plots of FDRs of enrichment analysis of top-ranked genes identified by EMDomics and SAM, using the "Investigate Gene Sets" function of the web-based GSEA tool provided by the Broad Institute. (a) q-values for the top 14 enriched Canonical and KEGG pathways in the 293 unique top-ranked genes. (b) q-values for the top 19 enriched oncogenic signatures in the 293 unique top-ranked genes.

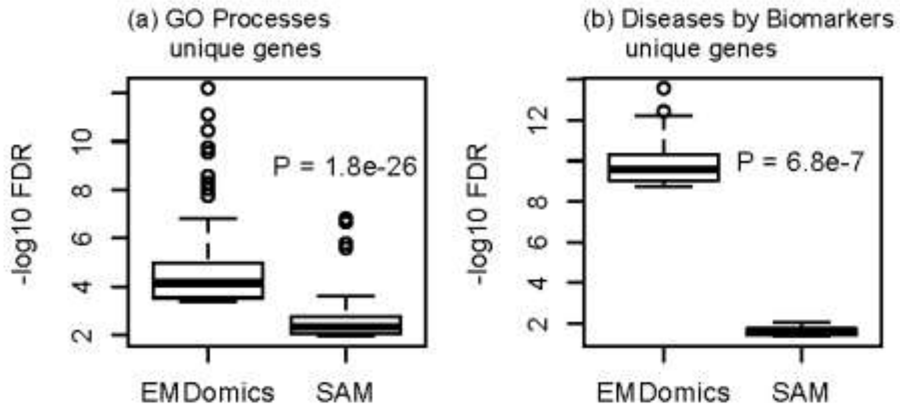

Fig. S12. Box plots of FDRs of enrichment analysis of top-ranked genes identified by EMDomics and SAM, using the MetaCore web-based enrichment analysis tool. (a) q-values for the top 100 enriched GO Processes in the 293 unique top-ranked genes. (b) q-values for the top 17 enriched Diseases by Biomarkers in the 293 unique top-ranked genes.

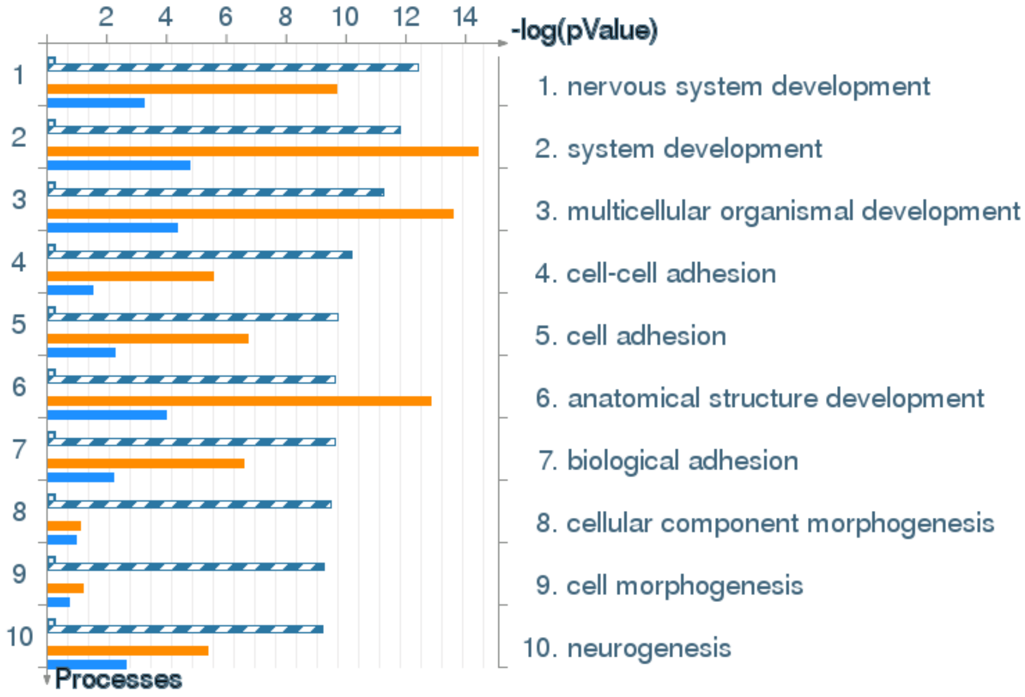

Fig. S13. Top 10 Gene Ontology (GO) cellular processes**.** The blue/white striped bar are for common genes among EMDomics significant genes and SAM top 475 genes, orange bars are for only EMDomics significant genes and blue bars are for only top SAM genes. Sorting is done for the 'common' set. For each of the top 10 gene sets, the EMDomics only genes (orange) are more enriched than the SAM only genes (blue).

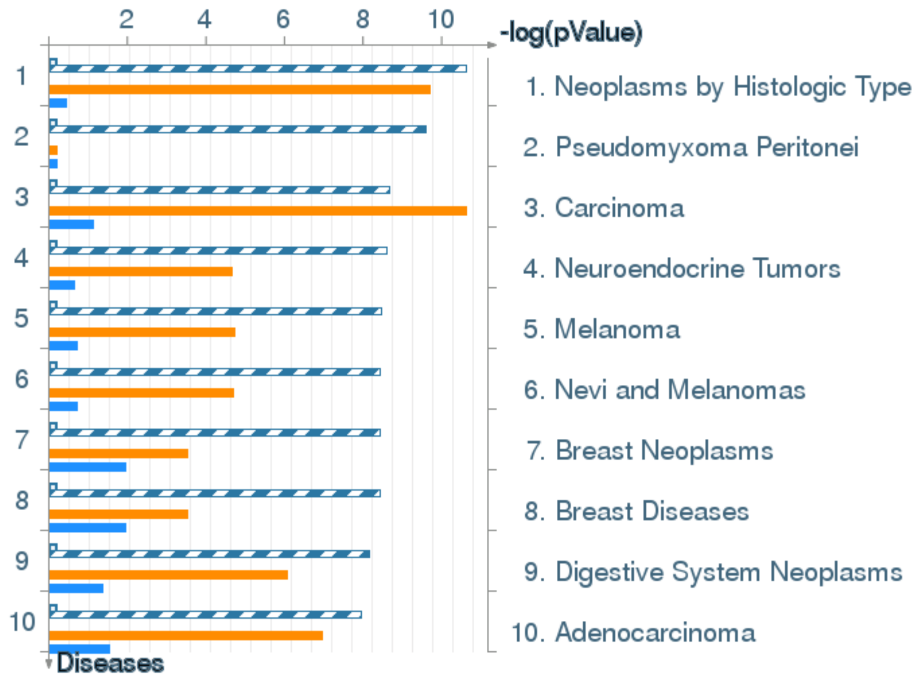

Fig. S14. Top 10 Diseases (by Biomarkers). The blue/white striped bars are for common genes among EMDomics significant genes and SAM top 475 genes, orange bars are for only EMDomics significant genes and blue bars are for only top SAM genes. Sorting is done for the 'common' set. For each of the top 10 gene sets, the EMDomics only genes (orange) are more enriched than the SAM only genes (blue).

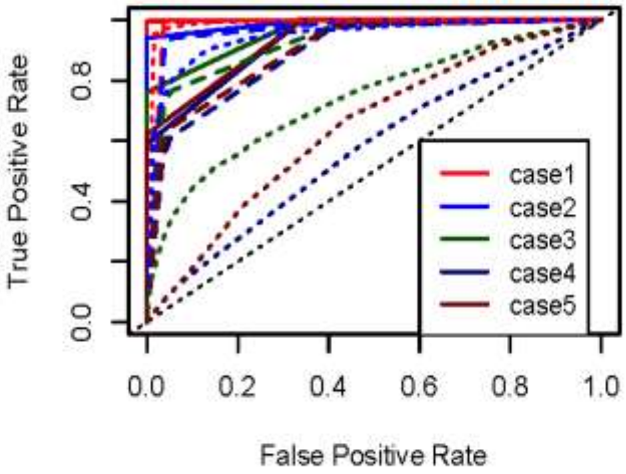

Fig. S15. ROC curves of KS, CVM and EMDomics for five cases with different level of heterogeneity in resistant samples, ranging from no intra-group heterogeneity (case 1) to significant intra-group heterogeneity (case 4 and case 5) as described in Table 1. Solid lines are for EMD, dashed lines are for CVM, and dotted lines are for KS.

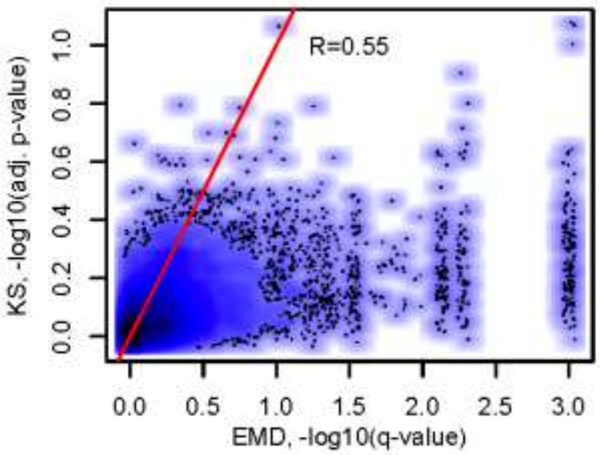

Fig. S16 q-value of EMDomics versus adjusted p-value of KS. q-values of zeros are set to 10^{-3}.

Supplementary tables

Table S1. Clinical information of the resistant and sensitive to cis-platinum in HG-SOC samples from TCGA

	**TCGA Cohort**		
	**Clinical Chemosensitive**	**Clinical Chemoresistant**	**Totals(All)**
**No. of patients**			
	234	97	331
**Age**			
**Mean,yrs[SD]**	58.1[11.42]	60.5[11.25]	58.7[11.41]
**Range**	26-87	38-87	26-87
**FIGO Stage**			
**I-II**	23	1	24
**III**	179	83	262
**IV**	31	14	45
**WHO Grade**			
**1**	3	0	3
**2**	34	9	43
**3**	192	86	278
**4 and X**	5	1	6
**Unknown**	0	1	1
**Surgical outcome**			
**Optimal(≤1cm)**	94	57	151
**Suboptimal (>1cm)**	56	26	82
**No Macroscopic disease**	60	10	70
**Unknown**	24	4	28

Table S2. The top 15 genes with the lowest q-value by EMDomics and the highest q-value by SAM

Gene	log FC	Limma p-value	Limma adj. p-value	SAM q-value	EMD	EMD q-value
PITX2	-0.0286	0.9221	0.9837	0.8369	3.4401	<0.001
GPC5	-0.0653	0.7822	0.9555	0.8084	2.3172	<0.001
EDN3	-0.2381	0.4676	0.8570	0.6697	2.3600	<0.001
CT45-6	0.5010	0.2952	0.7639	0.6432	2.6429	<0.001
PTN	-0.2078	0.4130	0.8329	0.6432	2.3779	<0.001
NLRP2	0.3133	0.2864	0.7578	0.6432	2.3424	<0.001
WDR69	0.3521	0.2083	0.7072	0.6171	2.5665	<0.001
RP13-36C9.6	0.5536	0.1959	0.7041	0.5944	2.8870	<0.001
COL11A1	0.4728	0.1753	0.6799	0.5944	2.4342	<0.001
MAGEC2	0.4316	0.1635	0.6675	0.5817	2.6773	<0.001
FLJ46266	0.4492	0.1726	0.6762	0.5817	2.3656	<0.001
HSPB3	0.3555	0.1138	0.6095	0.5403	2.2728	<0.001
FABP6	0.4944	0.1117	0.6073	0.5275	2.5839	<0.001
IGF2	0.5127	0.0821	0.5549	0.4775	2.6881	<0.001
OCA2	-0.2774	0.1704	0.6742	0.4775	2.3805	<0.001

Table S3. Significant genes by SAM with q-value< 0.05.

Gene	log FC	Limma p-value	Limma adj. p-value	SAM q-value	EMD	EMD q-value
SERTAD4	-0.6502	0.0003	0.1593	< 0.001	3.5475	< 0.001
LIPG	-0.7034	0.0000	0.1006	< 0.001	3.4718	< 0.001
MATN2	-0.6675	0.0000	0.0435	< 0.001	3.4378	< 0.001
GJB1	-0.6010	0.0001	0.1593	< 0.001	3.1161	< 0.001
LCTL	0.5154	0.0000	0.0435	< 0.001	2.6060	< 0.001
PGRMC1	-0.4542	0.0000	0.1189	< 0.001	2.2917	< 0.001
SLC27A6	-0.6946	0.0009	0.1890	0.0428	3.5220	< 0.001
SUSD4	-0.6197	0.0003	0.1593	0.0428	3.0827	< 0.001
PCDHB15	-0.4913	0.0006	0.1844	0.0428	2.4779	< 0.001
FZD4	-0.4791	0.0002	0.1593	0.0428	2.4127	< 0.001
PDZD2	-0.4476	0.0004	0.1593	0.0428	2.3821	< 0.001
EPHB3	-0.4647	0.0007	0.1858	0.0428	2.2892	< 0.001
MPZL2	-0.4511	0.0004	0.1593	0.0428	2.2692	< 0.001
SDF2L1	-0.4060	0.0004	0.1593	0.0428	2.1507	0.0053
KCTD1	-0.4345	0.0002	0.1593	0.0428	2.1273	0.0053
GCNT1	-0.4269	0.0004	0.1593	0.0428	2.1125	0.0053
FAM59A	-0.4171	0.0003	0.1593	0.0428	2.0597	0.0074
RMI1	-0.3745	0.0003	0.1593	0.0428	1.9241	0.0074
SEMA4D	-0.3307	0.0001	0.1593	0.0428	1.6813	0.0456
TACSTD1	-0.3195	0.0003	0.1593	0.0428	1.5971	0.0625
MSH6	-0.3097	0.0002	0.1593	0.0428	1.5506	0.0792
SPCS2	-0.2882	0.0002	0.1593	0.0428	1.4920	0.0952
ZNF592	-0.2915	0.0001	0.1532	0.0428	1.4101	0.1384

Table S4. Top 10 pathways and oncogenic signatures enriched by significantly differential expressed genes called by EMD

Canonical and KEGG pathways	q-value	Oncogenic signatures	q-value
REACTOME_CELL_CELL_JUNCTION_ORGANIZATION	3.26E-06	P53_DN.V1_DN	9.33E-14
REACTOME_CELL_JUNCTION_ORGANIZATION	3.26E-06	LEF1_UP.V1_DN	8.68E-10
REACTOME_ADHERENS_JUNCTIONS_INTERACTIONS	3.26E-06	BMI1_DN.V1_UP	1.15E-08
REACTOME_CELL_CELL_COMMUNICATION	1.35E-05	KRAS.600_UP.V1_UP	4.38E-08
PID_WNT_SIGNALING_PATHWAY	2.15E-03	KRAS.KIDNEY_UP.V1_UP	7.33E-08
REACTOME_GPCR_LIGAND_BINDING	4.38E-03	KRAS.600.LUNG.BREAST_UP.V1_UP	2.40E-07
REACTOME_PEPTIDE_LIGAND_BINDING_RECEPTORS	4.38E-03	PRC2_EZH2_UP.V1_DN	2.88E-07
REACTOME_EXTRACELLULAR_MATRIX_ORGANIZATION	4.78E-03	KRAS.BREAST_UP.V1_UP	5.74E-07
KEGG_PATHWAYS_IN_CANCER	4.90E-03	REACTOME_CELL_JUNCTION_ORGANIZATION	8.98E-07
KEGG_CELL_ADHESION_MOLECULES_CAMS	8.95E-03	PTEN_DN.V1_DN	9.38E-07

Gene sets come from the MSigDB (http://www.broadinstitute.org/gsea/msigdb/index.jsp)

Table S5. Top 10 oncogenic signatures enriched by significantly differential expressed genes called by EMD (475 genes) and the 475 top-ranked genes called by SAM.

Oncogenic signatures enriched by EMDomics significant genes	q-value	Oncogenic signatures enriched by 475 top-ranked SAM genes	q-value
P53_DN.V1_DN	1.17E-14	P53_DN.V1_DN	2.81E-07
LEF1_UP.V1_DN	1.09E-10	LEF1_UP.V1_DN	8.38E-06
BMI1_DN_MEL18_DN.V1_UP	1.44E-09	STK33_DN	2.14E-05
BMI1_DN.V1_UP	1.44E-09	NFE2L2.V2	3.08E-05
KRAS.600_UP.V1_UP	5.49E-09	P53_DN.V1_UP	3.09E-05
KRAS.KIDNEY_UP.V1_UP	9.18E-09	STK33_NOMO_DN	6.70E-05
KRAS.600.LUNG.BREAST_UP.V1_UP	3.01E-08	IL15_UP.V1_UP	1.02E-04
PRC2_EZH2_UP.V1_DN	3.61E-08	IL2_UP.V1_UP	1.02E-04
KRAS.BREAST_UP.V1_UP	7.19E-08	RAF_UP.V1_DN	1.02E-04
PTEN_DN.V1_DN	1.49E-07	E2F3_UP.V1_UP	1.02E-04

Table S6. Top 10 Canonical and KEGG Pathways enriched by significantly differential expressed genes called by EMD(475 genes) and the SAM's 475 top-ranked genes.

Canonical and KEGG pathways enriched by EMDomics significant genes	q-value	Canonical and KEGG pathways enriched by 475 top-ranked SAM genes	q-value
REACTOME_CELL_CELL_JUNCTION_ORGANIZATION	3.26E-06	KEGG_PATHOGENIC_ESCHERICHIA_COLI_INFECTION	1.96E-04
REACTOME_CELL_JUNCTION_ORGANIZATION	3.26E-06	KEGG_CELL_ADHESION_MOLECULES_CAMS	6.60E-03
REACTOME_ADHERENS_JUNCTIONS_INTERACTIONS	3.26E-06	KEGG_BASAL_CELL_CARCINOMA	8.82E-03
REACTOME_CELL_CELL_COMMUNICATION	1.35E-05	KEGG_PATHWAYS_IN_CANCER	1.20E-02
PID_WNT_SIGNALING_PATHWAY	2.15E-03	REACTOME_DEVELOPMENTAL_BIOLOGY	1.68E-02
REACTOME_GPCR_LIGAND_BINDING	4.38E-03	REACTOME_IONOTROPIC_ACTIVITY_OF_KAINATE_RECEPTORS	3.06E-02
REACTOME_PEPTIDE_LIGAND_BINDING_RECEPTORS	4.38E-03	PID_WNT_SIGNALING_PATHWAY	3.06E-02
REACTOME_EXTRACELLULAR_MATRIX_ORGANIZATION	4.78E-03	REACTOME_SLC_MEDIATED_TRANSMEMBRANE_TRANSPORT	3.06E-02
KEGG_PATHWAYS_IN_CANCER	4.90E-03	KEGG_O_GLYCAN_BIOSYNTHESIS	3.06E-02
KEGG_CELL_ADHESION_MOLECULES_CAMS	8.95E-03	REACTOME_DNA_STRAND_ELONGATION	3.06E-02

Table S7. Top 10 oncogenic signatures enriched by unique significantly differential expressed genes called by EMD (293) and unique SAM's top-ranked genes (293).

oncogenic signatures enriched by only EMDomics significant genes	q-value	oncogenic signatures enriched by only SAM's top-ranked genes	q-value
P53_DN.V1_DN	3.62E-09	STK33_DN	6.66E-05
KRAS.600.LUNG.BREAST_UP.V1_UP	3.95E-08	STK33_NOMO_DN	2.63E-04
BMI1_DN_MEL18_DN.V1_UP	1.18E-07	P53_DN.V1_UP	2.14E-03
KRAS.600_UP.V1_UP	1.77E-07	NFE2L2.V2	2.86E-03
KRAS.300_UP.V1_DN	6.98E-07	P53_DN.V1_DN	7.09E-03
PTEN_DN.V1_DN	6.98E-07	TGFB_UP.V1_DN	7.09E-03
BMI1_DN.V1_UP	6.98E-07	TBK1.DF_UP	1.43E-02
ESC_V6.5_UP_LATE.V1_UP	6.98E-07	CSR_LATE_UP.V1_UP	1.88E-02
PTEN_DN.V1_UP	6.98E-07	SRC_UP.V1_DN	2.05E-02
SNF5_DN.V1_DN	1.56E-06	GCNP_SHH_UP_LATE.V1_UP	2.07E-02

Table S8. Top 10 Pathways enriched by unique significantly differential expressed genes called by EMDomics (293 genes) and the unique top-ranked genes called by SAM (293 genes).

Canonical and KEGG pathways enriched by only EMDomics significant genes	q-value	Canonical and KEGG pathways enriched by only top SAM genes	q-value
REACTOME_PEPTIDE_LIGAND_BINDING_RECEPTORS	3.89E-04	KEGG_PATHOGENIC_ESCHERICHIA_COLI_INFECTION	2.59E-03
REACTOME_EXTRACELLULAR_MATRIX_ORGANIZATION	7.71E-04	REACTOME_DNA_STRAND_ELONGATION	2.08E-02
REACTOME_G_ALPHA_I_SIGNALLING_EVENTS	1.65E-03	REACTOME_DEVELOPMENTAL_BIOLOGY	2.08E-02
REACTOME_CELL_JUNCTION_ORGANIZATION	1.89E-03	KEGG_DNA_REPLICATION	2.51E-02
PID_SYNDECAN_1_PATHWAY	1.89E-03	REACTOME_PROCESSIVE_SYNTHESIS_ON_THE_LAGGING_STRAND	2.77E-02
REACTOME_GPCR_LIGAND_BINDING	1.89E-03	REACTOME_REGULATION_OF_SIGNALING_BY_CBL	3.62E-02
REACTOME_CELL_CELL_COMMUNICATION	1.89E-03	REACTOME_AXON_GUIDANCE	3.62E-02
REACTOME_ADHERENS_JUNCTIONS_INTERACTIONS	3.20E-03	REACTOME_LAGGING_STRAND_SYNTHESIS	3.62E-02
REACTOME_CLASS_A1_RHODOPSIN_LIKE_RECEPTORS	3.20E-03	REACTOME_IMMUNE_SYSTEM	4.52E-02
REACTOME_CELL_CELL_JUNCTION_ORGANIZATION	3.28E-03	KEGG_FC_GAMMA_R_MEDIATED_PHAGOCYTOSIS	4.63E-02

Table S9. Significant Pathways Maps enriched by significantly differential expressed genes called by EMDomics (475 genes).

Pathway Maps enriched by EMDomics significant genes	q-value	Pathway Map objects from the EMDomics significant gene list
Protein folding and maturation_Angiotensin system maturation \ Human version	6.38E-04	Angiotensin II, Angiotensin III, Angiotensin (2-10), Angiotensin (1-7), Angiotensinogen, Angiotensin (1-9), Angiotensin IV, Angiotensin I
Protein folding and maturation_Angiotensin system maturation \ Rodent version	7.64E-04	Angiotensin II, Angiotensin III, Angiotensin (2-10), Angiotensin (1-7), Angiotensinogen, Angiotensin (1-9), Angiotensin IV, Angiotensin I
Development_WNT signaling pathway. Part 2	1.00E-02	E-cadherin, Matrilysin (MMP-7), Tcf(Lef), Lef-1, Frizzled, ENC1, DKK1
Cell adhesion_ECM remodeling	4.63E-02	Matrilysin (MMP-7), MMP-1, LAMA4, IGF-2, MMP-13, Stromelysin-2
Development_TGF-beta-dependent induction of EMT via SMADs	4.63E-02	N-cadherin, E-cadherin, HMGA2, Lef-1, MKL1
PGE2 pathways in cancer	4.78E-02	COX-1 (PTGS1), Tcf(Lef), PKA-reg (cAMP-dependent), Amphiregulin, Lef-1, SLC21A2
